# Personalized prediction model generated with machine learning for kidney function one year after living kidney donation

**DOI:** 10.1038/s41598-025-02879-y

**Published:** 2025-07-01

**Authors:** Rikako Oki, Toshihio Hirai, Kazuhiro Iwadoh, Yu Kijima, Hiroyuki Hashimoto, Yasunori Nishimura, Taro Banno, Kohei Unagami, Kazuya Omoto, Tomokazu Shimizu, Junichi Hoshino, Toshio Takagi, Hideki Ishida, Toshihito Hirai

**Affiliations:** 1 Department of Organ Transplant Medicine, Tokyo Women’s Medical University, Shinjuku City, Tokyo Japan; 2 Department of Nephrology, Tokyo Women’s Medical University, Shinjuku City, Tokyo Japan; 3 Department of Urology, Tokyo Women’s Medical University, 8-1 Kawadacho, Shinjuku-ku, Tokyo, 162-8666 Japan; 4 Department of Transplant Surgery, Mita Hospital, International University of Health and Welfare, Minato City, Japan; 5 Department of Radiation Oncology, Tokyo Women’s Medical University, Shinjuku City, Tokyo Japan

**Keywords:** Kidney transplantation, Living donor, Machine learning, Kidney function post-donation, Prediction, Kidney, Health care

## Abstract

Living kidney donors typically experience approximately a 30% reduction in kidney function after donation, although the degree of reduction varies among individuals. This study aimed to develop a machine learning (ML) model to predict serum creatinine (Cre) levels at one year post-donation using preoperative clinical data, including kidney-, fat-, and muscle-volumetry values from computed tomography. A total of 204 living kidney donors were included. Symbolic regression via genetic programming was employed to create an ML-based Cre prediction model using preoperative clinical variables. Validation was conducted using a 7:3 training-to-test data split. The ML model demonstrated a median absolute error of 0.079 mg/dL for predicting Cre. In the validation cohort, it outperformed conventional methods (which assume post-donation eGFR to be 70% of the preoperative value) with higher R^2^ (0.58 vs. 0.27), lower root mean squared error (5.27 vs. 6.89), and lower mean absolute error (3.92 vs. 5.8). Key predictive variables included preoperative Cre and remnant kidney volume. The model was deployed as a web application for clinical use. The ML model offers accurate predictions of post-donation kidney function and may assist in monitoring donor outcomes, enhancing personalized care after kidney donation.

## Introduction

Careful screening of living donor candidates is critical to minimizing the risk of end-stage kidney disease (ESKD) and ensuring ongoing monitoring of renal function post-donation. Although rigorously screened living kidney donors were traditionally thought to have comparable risks of mortality and ESKD to the general population^1^, they may face a higher risk of ESKD compared to matched healthy non-donors^2^ Additionally, selection criteria for living donors have broadened to include medically complex individuals, such as those with advanced age, hypertension, obesity, or lower estimated glomerular filtration rate (eGFR)^3^ These evolving trends underscore the urgent need for more precise and individualized prediction models to assess and monitor postoperative renal function in this population.

Post-donation eGFRs are typically reach approximately 60–70% of the pre-donation levels, attributed to compensatory hypertrophy of the remaining kidney^4^ Grams et al. developed an online risk tool to estimate the long-term risk of ESKD for living kidney donor candidates, using meta-analyzed risk associations from seven general population cohorts^5^ Several reports have proposed predictive factors for post-donation kidney function that included donor age, sex, race, body mass index (BMI) and preoperative computed tomography (CT) volumetry of kidney^6–9^ These were based on observational study and traditional statistics which aims to identify significant risk factors among explanatory variables through model-driven regression using linear models^10^.

Traditional regression models that fit data to pre-defined models are directly meaningful to clinicians, though it relies on liner predefined models and are constrained by strict assumptions. In contrast, symbolic regression (SR) via genetic programming (GP) is a machine learning (ML) algorithm where the goal is discovering an explicit mathematical formula that best describes a given dataset from the vast function space, enabling it to uncover complex, non-linear interactions directly from the data^11^ Genetic programming (GP) explores both the structure of the model and its parameters. This evolutionary methodology is based on the principles of mutation and natural selection, mirroring the way organisms evolve and adapt to their environments. In SR via GP, innumerable mathematical formulas evolve to fit the given dataset, progressively yielding better ones that can predict the target value from the explanatory variables. In the previous study, Ueno et al. utilized SR via GP to evaluate pathological factors associated with the development of glomerular hypertrophy (GH)^12^ From a set of 60 variables, the SR model identified key factors such as inflammation, vascular damage, and obesity as significant predictors, while eGFR was ranked low (46th out of 60). This finding highlights the ability of SR to distinguish morbid GH from adaptive hypertrophy due to nephron loss. Collectively, these results suggest that SR can evaluate variables in an unbiased manner, providing valuable insights into the underlying mechanisms.

The objective of this study was to develop a machine learning model for more accurate and personalized prediction of post-donation kidney function. We employed an SR model to predict post-donation creatinine (Cre) values using preoperative variables, including CT volumetry data for excised and non-excised kidney volumes. Given that excess visceral fat and reduced skeletal muscle mass—characteristic of sarcopenia—are recognized risk factors for the development of chronic kidney disease (CKD)^13–15^, we also implemented CT volumetry for visceral fat and skeletal muscle. The resulting model was integrated into a user-friendly interface to facilitate clinical application and improve decision-making in transplant practice.

## Results

### Patient’s background

The clinical characteristics and laboratory data of the patients in the study cohorts are summarized in Table 1. All the participants were Asian. The mean donor age was 59.9 years, and 35% were male. The median baseline Cre level before donation was 0.7 mg/dl. There were no significant differences in the variables between the training and the validation cohort.

**Table 1:** Comparison between training cohort and validation cohort.

Variables	All (*N* = 204)	Training cohort (*N* = 143)	Validation cohort (*N* = 61)	*p*
Age at donation (y.o)	59.9 ± 8.78	60.3 ± 8.70	59.0 ± 8.96	0.363
Male [n (%)]	71 (34.8)	53 (37.1)	18 (29.5%)	0.338
Body weight (kg)	56.0 (51.0, 63.9)	57.0 (51.0, 64.0)	53.0 (51.0, 61.0)	0.098
Hight (cm)	159.0(154.5, 165.0)	160 (155, 165)	159 (154, 164)	0.698
BMI	22.4 (20.4, 24.2)	22.6 (20.6,24.4)	21.9 (20.2, 23.2)	0.053
Systolic blood pressure (mmHg)	129.9 ± 19.9	130.7 ± 19.7	127.9 ± 20.3	0.357
Diastolic blood pressure (mmHg)	76.7 ± 14.3	77.4 ± 13.0	75.1 ± 17.0	0.284
smoking [n (%)]	58 (28.4)	42 (29.4)	16 (26.2)	0.736
History of CVD [n (%)]	4 (2.0)	3 (2.1)	1 (1.6)	1.000
Antihypertensive agents [n (%)]	40 (19.6)	27 (18.9)	13 (21.3)	0.703
Lipid-lowering agents [n (%)]	21 (10.3)	12 (8.4)	9 (14.8)	0.209
uric acid lowering agents [n (%)]	4 (2.0)	4 (2.8)	0 (0)	0.319
WBC (/µl)	4955 (4265, 6055)	4930 (4270, 5995)	5190 (4240, 6180)	0.490
Hb (g/dl)	13.7 ± 1.24	13.8 ± 1.27	13.6 ± 1.18	0.468
Platelet (*10^3^/µl)	21.7 (18.2, 25.4)	22.0 (18.3, 25.3)	21.0 (18.2, 26.0)	0.846
Cre (mg/dl)	0.70 (0.62, 0.81)	0.69 (0.62, 0.84)	0.70 (0.62, 0.78)	0.612
eGFR (ml/min/1.73m^2^)	71.9 (65.1, 80.3)	71.4 (65.0, 80.2)	72.7 (65.1, 80.2)	0.650
BUN (mg/dl)	13.8 (11.2, 15.7)	13.8 (11.2, 15.8)	13.8 (11.7, 15.6)	0.646
Na (mEq/L)	142 (141,143)	142 (141, 143)	141 (141, 143)	0.417
K (mEq/l)	4.2 (4.0, 4.4)	4.2 (4.0, 4.4)	4.2 (4.0, 4.4)	0.917
AST [IU/l]	20.0 (17.0, 24.0)	20.0 (17.0, 24.5)	20.0 (17.0, 24.0)	0.849
ALT [IU/l]	17.0 (13.0, 22.0)	17.0 (13.0, 22.0)	17.0 (14.0, 23.0)	0.583
TP (g/dl)	7.24 ± 0.36	7.24 ± 0.37	7.24 ± 0.34	0.989
CRP (mg/dl)	0.05 (0.04, 0.10)	0.05 (0.04, 0.11)	0.05 (0.04, 0.10)	0.460
T.chol (mg/dl)	211 (187, 233)	213 (186, 233)	205 (190, 233)	0.521
UA (mg/dl)	4.80 (4.10, 5.90)	4.90 (4.10, 5.90)	4.70 (4.10, 5.90)	0.524
Glucose (g/dl)	98.0 (92.0, 104)	98.0 (92.0, 104)	98.0 (92.0, 107)	0.905
HbA1C (%)	5.70 (5.50, 5.90)	5.70 (5.50, 5.90)	5.70 (5.50, 5.90)	0.772
Proteinuria (+)	2 (1.0)	1 (0.7)	1 (1.6)	0.510
Urinary occult blood (≥+)	14 (6.9)	11 (7.7)	3 (4.9)	0.561
Cre at 1-year post-donation (mg/dl)	1.06 (0.93, 1.23)	1.06 (0.93, 1.26)	1.06 (0.91, 1.18)	0.378
Results of CT findings				
Volume of excised kidney (ml)	143 (126, 161)	148 (129, 163)	140 (118, 157)	0.133
Volume of non-excised kidney(ml)	137 (120, 156)	138 (121, 159)	133 (114, 152)	0.165
Area psoas muscle at L3(cm^2^)	11.3 (9.01,15.0)	11.5 (9.13, 14.9)	10.7 (8.92, 15.2)	0.501
Area skeletal muscle at L3(cm^2^)	98.5 (85.8, 126)	100.5 (87.0, 126)	92.1 (82.5, 120)	0.139
Area visceral fat(cm^2^)	86.4 (56.0, 125)	82.1 (58.2, 126)	90.2 (50.9, 115.7)	0.528
Area visceral Fat at L3 (cm^2^)	77.2 (38.9, 127)	77.2 (46.1, 135)	81.1 (38.2, 118)	0.516

Continuous data are presented as mean ± SD or median (IQR): CVD, cardiovascular disease; WBC, white blood cell; Hb, hemoglobin; Cre, creatinine; eGFR, estimated glomerular filtration rate; BUN, blood urea nitrogen; Na, sodium; K, potassium; AST, aspartate aminotransferase; ALT, alanine aminotransferase; TP, total protein; CRP, C-reactive protein; T.chol, total cholesterol; UA, uric acid; HbA1C, hemoglobinA1C.

### CT volumetry data

There were no significant differences in CT volumetry data between training cohort and validation cohort (Table 1). The correlation between CT volumetry data and preoperative Cre level was investigated using Pearson’s correlation coefficient (r). The analysis revealed moderate positive correlations between area of psoas muscle/skeletal muscle/visceral fat and pre-operative Cre level (Supplementary table 1A). Significant positive correlations were observed between CT volumetry data and pre-operative body weight (Supplementary table 1B).

### Correlation between explanatory variables and post-donation cre level

All correlation coefficients between preoperative data and Cre level at 1 year post-donation are presented in Supplemental Fig. 1. Additionally, the point-biserial correlation coefficients (r values) for the top 10 variables with the strongest positive correlations are summarized in Table 2. As with the pre-operative Cre level, the Cre level at 1 year post-donation demonstrated a positive correlation with preoperative skeletal muscle volume (*r* = 0.62, *p* < 0.001), along with weak positive correlations with the psoas muscle and visceral fat volumes.

**Table 2:** Correlation between pre-operative variables and creatinine level at 1 year post-donation.

Variables	*r* (95% CI)	*p*
Cre	0.84 (0.80–0.88)	< 0.001
Male	0.65 (0.56–0.72)	< 0.001
Area skeletal muscle at L3	0.62 (0.53–0.70)	< 0.001
Body weight	0.57 (0.47–0.65)	< 0.001
UA	0.51(0.40–0.60)	< 0.001
Height	0.47 (0.35–0.57)	< 0.001
Area visceral Fat at L3	0.46 (0.34–0.56)	< 0.001
Hb	0.40 (0.27–0.51)	< 0.001
Area visceral fat	0.37 (0.24–0.48)	< 0.001
Area psoas muscle at L3	0.25 (0.12–0.37)	< 0.001

Cre, creatinine. UA, uric acid. Hb, hemoglobin.

### Analysis of predictive variables for Cre level post-donation using ML

**Fig. 1:**
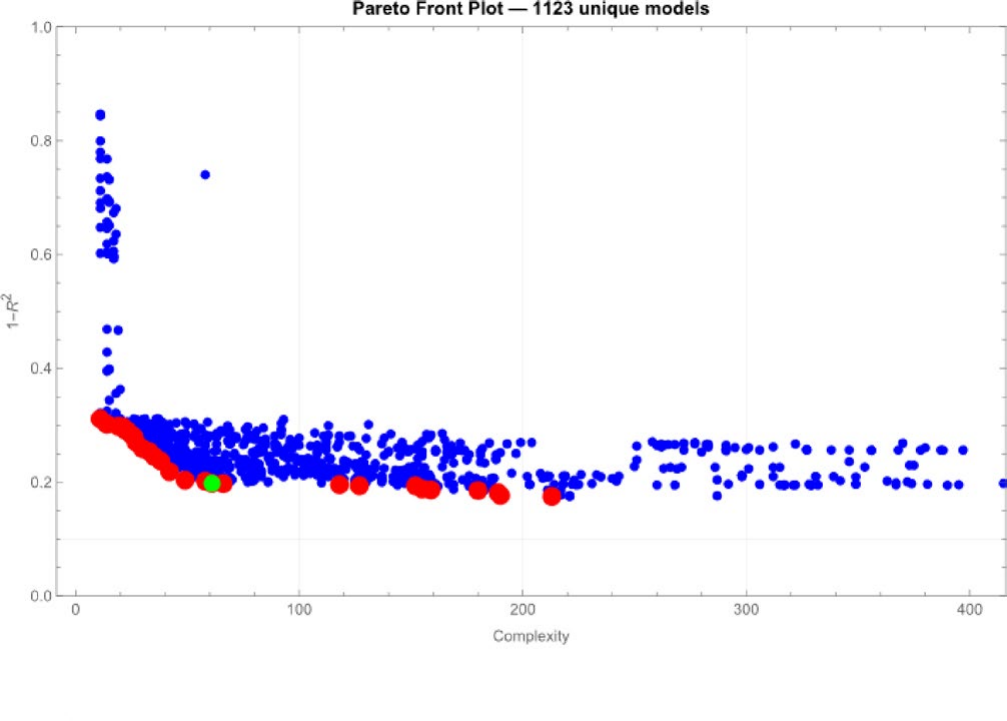
Distribution of functions generated using symbolic regression via genetic programming within the function space. This figure illustrates the distribution of complexities and errors of generated models in a function space. The horizontal axis represents complexity, while the vertical axis indicates error. Each dot corresponds to a single generated model. Red dots represent models on the Pareto Front, and the green dot marks the model positioned at the uppermost and rightmost edge of a rectangle used to select appropriate models that strike a balance between underfitting and overfitting.

**Fig. 2:**
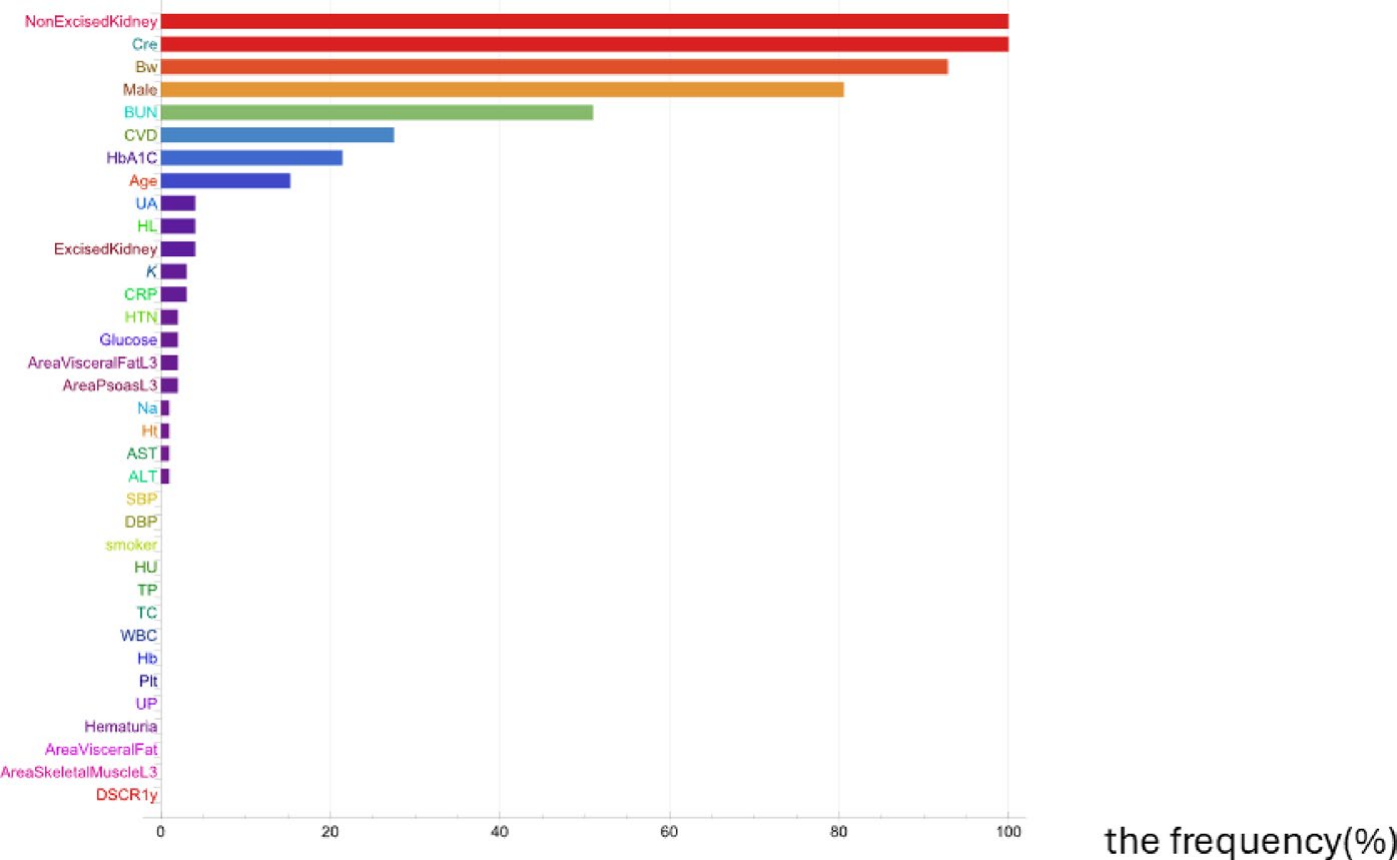
Frequently of variables utilized in the selected models. The frequency of variables used in the selected models is shown in descending order. The horizontal axis represents the frequency of appearance. Cre, creatinine; Bw, body weight; BUN, blood urea nitrogen; CVD, cardiovascular disease; HbA1C, hemoglobinA1C; UA, uric acid; HL, hyperlipidemia (lipid-lowering agents); K, potassium; CRP, C-reactive protein; HTN, hypertension; Na sodium; Ht, height; AST, aspartate aminotransferase; ALT, alanine aminotransferase; SBP, systolic blood pressure; DBP, Diastolic blood pressure; HU, hyper uricemia (uric acid lowering agents); TP, total protein; TC, total cholesterol;

By using 34 explanatory variables, Data Modeler automatically generated 1123 models that calculate Cre level at 1-year post-donation. Figure 1 show their distribution in the function space. We selected 98 models with lower complexities and lower 1-R^2^ in each epoch to limit the number of models to 9% of all generated models. We ranked the frequencies of all explanatory variables that were used in the selected models (Fig. 2). The selected factors for generating models included age, male, BW, the history of CVD, BUN, Cre, HbA1C, and the volume of the non-excised kidney.

### Creating a predictive model for Cre level at 1-year

**Fig. 3:**
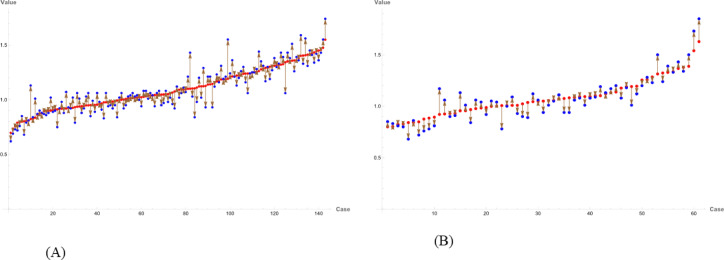
Relationship between calculated and measured creatinine values in (**A**) training cohort and (**B**) validation cohort. Red dots represent the calculated creatinine values obtained from the optimized DKF model. Blue dots represent the measured creatinine values at 1 year post-donation. Arrows indicate the discrepancies between the calculated and measured values.

To establish an optimized model for predicting Cre levels 1 year post-donation, ensemble learning was performed using the bagging method, which calculates the trimmed means of the selected models. The formula for the optimized DKF model, incorporating key variables extracted from the developed models, is provided in Supplemental Document 1. The R^2^, RMSE, and MAE values between the predicted Cre levels generated by the optimized DKF model and the measured values were 0.72, 0.1, 0.073 mg/dl mg/dL in the training cohort (Fig. 3A) and 0.69, 0.1, and 0.079 mg/dl in the validation cohort (Fig. 3B), respectively.

### The impact of each variable in the generated formula

We investigated factors that were used in the generated formula to determine which factors had higher impact on the target. A simulation was performed by changing each variable within its range, while the influence of all other variables was averaged out. Table 3 lists the 8 most significant driver variables that influenced the result. Pre-Cre level and the volume of the non-excised kidney were the most influential factors for Cre levels at 1 year, with high partial dependence estimate. Supplementary Fig. 2 illustrates the extent to which the target Cre value changes as the top 3 driver variables are varied.

**Table 3:** The changes in the Cre level as each explanatory variable changes from its minimum to its maximum while the influence of all other variables was averaged out.

	Variables	Partial Dependence Estimate	95% Confidential Interval
1	pre Cre level	0.69	0.67–0.72
2	volume of non-excised kidney	0.44	0.36–0.51
3	body weight	0.28	0.22–0.34
4	Male	0.09	0.03–0.15
5	BUN	0.06	-0.01-0.13
6	Pre HbA1C	0.02	-0.05-0.09
7	Age at donation	0.02	-0.05-0.09
8	History of CVD	0.01	-0.05-0.08

Cre, creatinine; BUN, blood urea nitrogen; HbA1C, hemoglobinA1C; CVD, cardiovascular disease.

### The development of a sparse model

Based on the above analysis, several valuables incorporated in the model have small impact on the estimated eGFR. To enhance user-friendliness in clinical settings, we developed a simplified prediction formula (referred to as the sparse DKF model), where variables with partial dependent estimate smaller than 0.1 were replaced with their median values. Specifically, age, BUN and HbA1C were fixed at their median values, while the history of CVD was set to its mode, which was 0. The key driving factors for this simplified model included sex (male), body weight (BW), Pre-Cre, and the volume of the non-excised kidney. The developed model has been uploaded to GitHub and implemented as a web application for convenient calculations, accessible at the following address: [https://donorcrcalculatoren-89wgze554kd9n2yjkcyubb.streamlit.app/].

### Verification of accuracy of developed models

To assess the accuracy of the developed models, we calculated the R^2^, RMSE, and MAE for the values predicted by the optimized DKF model, the sparse DKF model, and the conventional DKF model, comparing them to the measured eGFR values in the validation cohort (*N* = 61). (Table 4) The optimized DKF model achieved the highest R^2^ and the lowest RMSE and MAE, indicating minimal prediction error and high predictive accuracy. The sparse DKF model, designed with clinical practicality in mind, also demonstrated significantly better predictive accuracy compared to the conventional DKF model. Consequently, both the optimized DKF model and the sparse DKF model showed superior data fit and can be considered more reliable than the conventional DKF model.

**Table 4:** R-squared, RMSE and MAE in comparison of the predicted values by the optimized DKF model/the sparse DKF model/ the conventional DKF model and the observed eGFR values.

Model	*R*-squared	Root Mean Squared Error	Mean Absolute Error
The optimized DKF model	0.58	5.27	3.92
The sparse DKF model	0.52	5.36	4.17
The conventional DKF model	0.27	6.89	5.80

DKF, donor’s kidney function.

**Fig. 4:**
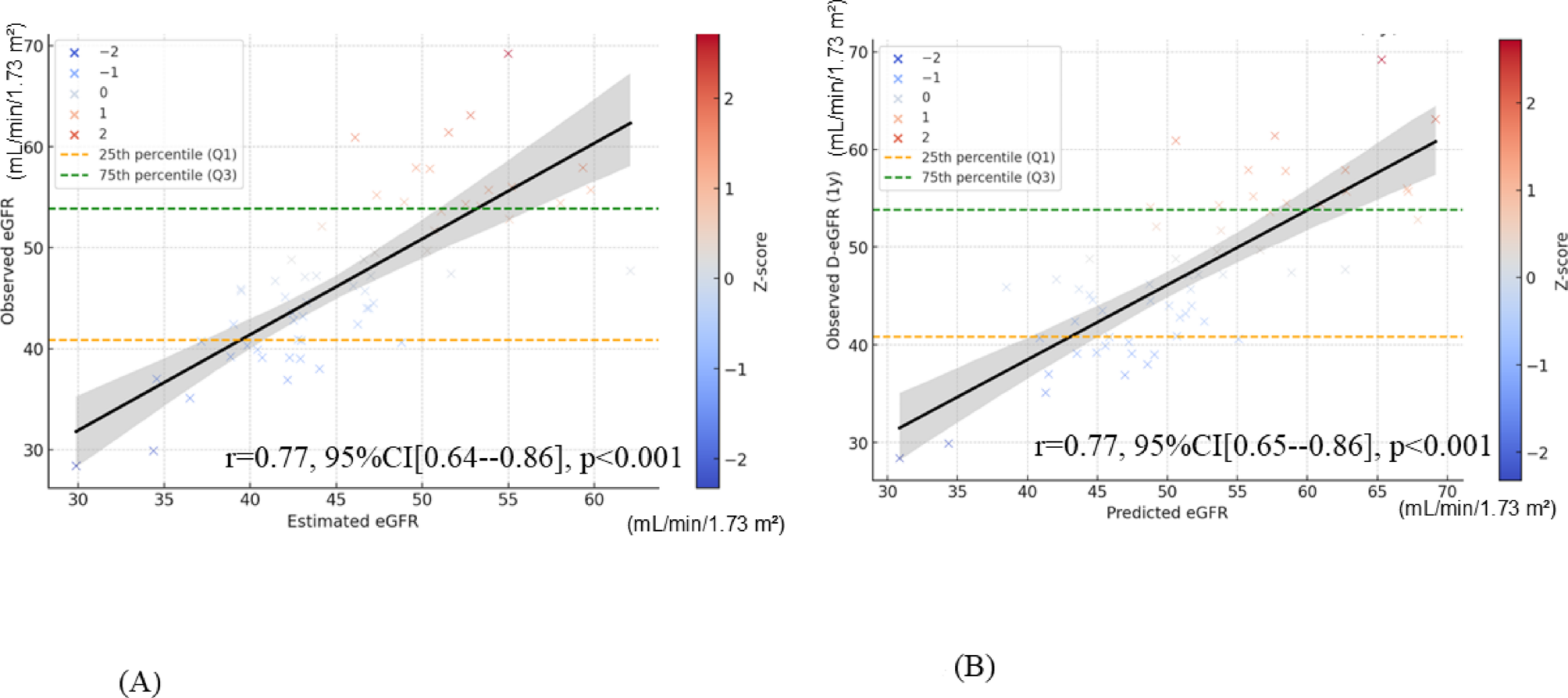
The correlation graphs with Z-score between observed eGFR and estimated eGFR. The plots show the observed eGFR at 1 year post-donation (Y-axis) versus the estimated eGFR (X-axis) using the optimized DKF model (**A**) and the conventional DKF model (**B**). The color scale represents the Z-score. The green dashed line indicates the 75th percentile, while the orange dashed line indicates the 25th percentile.

Next, we analyzed the correlation between the predicted values from the optimized DKF model and the measured eGFR values (Fig. 4A), as well as the predicted values from the conventional DKF model and the measured eGFR values (Fig. 4B). Three cases with |Z| ≥ 2 were identified, and these cases completely overlapped between the optimized DKF model and the conventional DKF model. Supplementary Table 2 compares the patient characteristics among inliers (-2 < Z < 2), high outliers (Z > 2), and low outliers (Z < -2) in the prediction of Cre at 1 year post-donation. Although the small sample size limits statistical interpretation, cases with higher preoperative Cre levels appeared more likely to be classified as outliers in both the optimized DKF model and the conventional DKF model.

## Discussion

In the present study, we generated a predictive model for Cre level at 1-year post-donation, using the SR via GP technique, which is one of the ML techniques. The developed model, referred to “the optimized DKF model” by ML was found to have higher accuracy, demonstrating higher R^2^ values and lower RMSE and MAE, compared to the conventional DKF model which assumed eGFR post-donation was 70% of the preoperative value. Preoperative Cre level and the volume of the non-excised kidney were identified as the most influential predictive factors for Cre levels at 1 year post-donation. Predicting the kidney function post-donation will facilitate more rigorous management and help achieve optimal post-transplant outcomes.

Statistical analysis revealed that there was a moderate correlation between the skeletal muscle volume and Cre levels at 1 year, as well as between the visceral fat volume and Cre levels at 1 year. The volume of non-excised kidney was not corelated to Cre level post-donation. Approximately 95% of the human body’s total creatine is located in skeletal muscle and serum Cre can be served as a surrogate marker of skeletal muscle mass even in CKD patients^16^ The previous study revealed that visceral adipose tissue detected by CT scan was associated with CKD when defined using cystatin C estimating equations but not when using a Cre-based estimating Eq. ^17^ On the other hand, area of skeletal muscle and area of visceral fat were not identified as influential factors in the ML analysis. Volume of non-excised kidney was found to be a factor affecting Cre level at 1-year. The larger the volume of the non-excised kidney, the lower the Cre levels at 1 year tended to be. (Supplementary Fig. 2A) Shimada et al. demonstrated that body surface area-adjusted preserved kidney volume calculated by the 3D reconstructed image was an independent risk factor for > 30% reduction of eGFR at 1-year post-donation (odds ratio, 0.93)^9^, which aligned with our findings. It is well known that adaptive hyperfiltration after donor nephrectomy is attributable to hyper perfusion and hypertrophy of the remaining glomeruli^18^ The increases in single-kidney renal plasma flow, cortical volume, and GFR continue through to the late post-donation period^18^ From the perspective of donor outcomes, it is suggested to determine which side of kidney to be excised based on the volume of the remaining kidney.

As previously mentioned, the post-donation eGFRs are approximately 60–70% of the pre-donation values based on past reports^4^ To assess the generated model’s goodness-of-fit, we compared its R^2^, RMSE, and MAE values to those of the conventional prediction method. The optimized DKF model showed higher explanatory power and accuracy for predicting Cre level at 1 year compared to the conventional method. The eGFR was reported to increase by + 0.35 ml/min/ 1.73 m^2^ per year from 6 weeks post-donation onward, with this increase leveling off by the fifth year^19^ It is reported that increase in GFR can begin as early as 8 h post-donation, however, this acute compensation is less efficient in older donors^20^ Thus, predicting Cre levels at 1-year by ML may be more beneficial than the conventional prediction method in clinical practice. However, outliers also exist in the optimized DKF model. Since the cases with pre-operative Cre values deviating from the median were likely to become outliers, the prediction may not be applied in the cases with extreme Cre level before the donation.

Then, how should this model be applied in clinical practice? While the rate of ESKD development among living donors is exceedingly low, it is well known that kidney donors had an increased risk of ESKD, compared with matched healthy nondonors^2^ Massie et al. measured eGFR at 6 months post-donation to estimate the 15-year cumulative incidence of ESKD, using data from 71,468 living kidney donors^21^After adjusting for age, race, sex, BMI, and biological relationship, each 10 mL/min/1.73 m² decrease in eGFR at 6 months post-donation was associated with a 28% higher risk of ESKD^21^ Longitudinal monitoring of post donation kidney function is expected to enhance risk assessment in living kidney donors. Thus, kidney function at 1-year post donation may serve as a surrogate marker for long-term risk of ESKD in living kidney donors. Ensuring the safety of living kidney donation is a top priority, and transplant physicians have a responsibility to provide careful post-nephrectomy monitoring, even when donors fully understand and accept the associated risks. It is meaningful to predict Cre levels at 1-year post-donation, to determine the follow-up intervals and management strategy. Additionally, this model is also valuable during pre-donation discussion with prospective living donors, as it provides an individualized estimate of kidney function at 1-year post-donation, thereby helping candidates to better visualize and understand their expected outcomes. Therefore, predicting kidney function at 1-year post donation is critically important for pre-donation counseling, and helps tailor post-donation monitoring strategies.

This model was developed by analyzing the characteristics of donors who were ultimately selected for kidney donation, excluding those who did not meet the criteria for a living kidney donor. Therefore, it is essential to note that even if a good Cre level was expected by the generated model, it does not guarantee the safety of prognosis for cases which don’t meet the criteria for a living kidney donor. It is important to adhere to the guidelines for evaluating eligibility for a living kidney donor. How to approach to donor selection has been discussed nowadays. While the Kidney Disease Improving Global Outcomes Guideline recommends the use of fixed cutoffs^22^, age and sex- based GFR cutoffs are commonly used in the British Transplantation Society, the European Renal Best Practice, and the Canadian Society of Transplantation, considering kidney function decline with healthy aging^23–25^ It is challenging to judge whether older candidates’ kidney function is appropriate for their age because they tend to be more medically complex. In such cases, which include so-called marginal donors, the prediction of kidney function post-donation by the generated model might be useful in planning follow-up. In cases where a donor is acceptable for kidney donation but is predicted to have poor post-donation kidney function, careful follow-up should be warranted after donation. Additionally, as non-excised kidney volume was an influential factor for predicting Cre level at 1-year, it is possible to simulate the impact on kidney function based on CT imaging data. It can be used as a reference to consider the impact on kidney function depending on which kidney will be donated. Overall, although the optimized DKF model/the sparse DKF model cannot be used directly for donor selection, it might be helpful as a reference for donor selection, for simulating kidney function post-donation, and for planning follow-up.

We acknowledge there are limitations in our study. First, this model was generated by data set from a single institution with a small sample size. According to the report by Guyon et al.^26^, even with only a few dozen samples, having a lot of features can make it possible to separate the training data perfectly with a linear classifier. We increased the feature dimensionality by adding variables, including CT volumetry, to address the small sample size. There might be more other factors affecting kidney function after donation beyond our dataset, however, increasing the number of features also increase the risk of overfitting and data noise^26^ Therefore, data validation using external data set is mandatory in the future study. Second, the formula developed in this study was designed solely to predict post‑donation kidney function, not to determine which kidney to remove. In fact, CT volumetry was not used for evaluating split renal function in this cohort. Habbous et al. performed a meta‑analysis of 19 studies (*n* = 1,479) and found that CT volumetry shows a moderate correlation with split renal function by nuclear renography (pooled Pearson’s *r* = 0.74; 95% CI, 0.61–0.82), yet its ability to detect a clinically meaningful between‑kidney function difference of ≥ 10% is limited: sensitivity 35%, specificity 88%, overall agreement 78%, and a false‑negative rate of approximately 14%.^27^ Although selection bias may have influenced these findings (since renography was often only performed in donors with marked size asymmetry), CT volumetry measures kidney volume alone and does not account for factors affecting true renal function—such as nephron density, scarring, or other underlying pathology. Therefore, when a significant split renal function disparity is suspected, nuclear renography remains the recommended evaluation method.

Finally, strictly speaking, the model can only be applied at the time a candidate presents for donation, because the blood pressure data used in the process of making model, was taken on the day of admission for donor nephrectomy. All other clinical factors, including CT findings, were obtained during the donor evaluation. However, in both the optimized DKF model and the sparse DKF model (a simplified model), blood pressure was not selected as a factor. Therefore, it may still serve as a useful reference during donation discussions with prospective living donors.

In conclusion, the ML model achieved an effective predictive performance for predicting Cre level post-donation. It is meaningful for transplant physicians to pay sufficient attention to the care for donors after donation. Applying ML techniques to the clinical field has the potential to lead to better healthcare for patients.

## Materials and methods

### Patients and data collection

We identified 204 patients who underwent enhanced CT prior to donor nephrectomy for living kidney transplantation at Tokyo Women’s Medical University Hospital between January 2012 and December 2016. Patients who were lost to follow-up after discharge or whose follow-up period after nephrectomy was less than one year were excluded. This study was approved by the Institutional Review Board of the Tokyo Women’s Medical University Hospital (#2021 − 0122). Since this is a retrospective study, informed consent was waived by the Institutional Review Board of the Tokyo Women’s Medical University Hospital. All methods of research procedures were performed in accordance with the Declaration of Helsinki.

Basic information about the patients was obtained from donor medical records within 3 months before donation, including age, sex, medications for hypertension, hyperlipidemia, and hyperuricemia, smoking habits, systolic/diastolic blood pressure, body mass index, and laboratory data. The laboratory data comprised white blood cell (WBC) count, hemoglobin (Hb), platelets, AST (aspartate aminotransferase), ALT (alanine aminotransferase), blood urea nitrogen (BUN), serum creatinine (Cre), estimated glomerular filtration rate (eGFR), sodium (Na), potassium (K), total protein (TP), C-reactive protein (CRP), uric acid (UA), hemoglobin A1c (HbA1c), total cholesterol (T. Chol), urinary protein, and urinary occult blood as qualitative tests. Blood pressure was measured with a brachial sphygmomanometer.

### Donor selection

Our donor selection is conducted in accordance with the Japanese criteria for living donor kidney transplantation. (Japanese donor guideline written in Japanese, https://cdn.jsn.or.jp/guideeline/pdf/Donor-guidelines.pdf, Supplementary Table 3A) For the marginal donor in our facility, who do not meet the Japanese standard criteria, we adopted our own marginal donor criteria. (Supplementary Table 3B)^28^ In our facility, donor candidates diagnosed with diabetes mellitus or suspected diabetes mellitus are excluded. (Criteria: fasting blood sugar ≤ 126 mg/dl and HbA1C < 6.2%, oral hypoglycemic agent is not allowed.)

For surgical assessment for determining which kidney to remove, CT volumetry was not used in the actual donor selection; it was performed retrospectively. If there was a clear difference in kidney size on CT, a renogram was performed. If the renogram showed no functional issues in either kidney, the decision on which kidney to donate was made based on the number and anatomical structure of the blood vessels.

### CT volumetry

Preoperative dynamic CT was performed using 64-,80- or 320-multidetector CT scanners (Aquilion 64, Aquilion Prime SP, Aquilion ONE; Canon Medical Systems Corporation, Otawara, Tochigi, Japan). Iodinated contrast media (Iohexol, Omnipaque 300; GE Healthcare Pharmaceutical Diagnostics, Tokyo, Japan) was injected using a power injector following an unenhanced CT scan. The nephrographic phase images were transferred and analyzed retrospectively using dedicated software (SYNAPSE VINCENT; FUJIFILM Corporation Tokyo, Japan). The software semi-automatically generated a 3D model of the kidney by stacking 1-mm axial slices and calculated the kidney’s volume (Supplementary Fig. 3A, B). Whole-body skeletal muscle mass was estimated from the trunk muscle area at the L3-4 level which is known to be highly correlated with the total body skeletal muscle volume (Supplementary Fig. 3C)^29^ As for the visceral fat area at the naval level, a single axial slice at the L3-L4 intervertebral space was chosen for analysis, as it is frequently used as a surrogate for abdominal adiposity (Supplementary Fig. 3C)^30^.

### Evaluation of renal function

Renal function, measured by serum Cre levels, was evaluated pre-donation and at 1-year post-donation. The pre-donation serum Cre level was defined as the most recent result obtained within 3 months before donation. Serum Cre level at 1-year post-donation was employed as the primary outcome for this study. The eGFR was calculated using a formula specific to Japanese patients with CKD (eGFR [mL/min/1.73 m^2^] = 194 x Serum creatinine(-1.094) × Age(-0.287) × 0.739 [if female])^31^.

### Statistical analysis

Continuous data were described as mean ± standard deviation or median (interquartile range). Unpaired t-test or Mann–Whitney U test was used to compare continuous variables. The chi-square test or Fisher’s exact test was used to compare the categorical variables. A Pearson’s correlation test was performed on correlation between clinical factors and Cre level at 1-year post donation. A Pearson’s correlation test was also performed to evaluate the correlation between the eGFR predicted by the generated/conventional model and the observed eGFR measured in practice. Values for which *p* was less than 0.05 were inferred as significant.

### Model generation

The data were randomly divided into a training and a validation data in a 7:3 ratio. By using training data, we conducted SR via GP to construct machine learning models for predicting Cre levels at 1-year post-donation using Data Modeler version 9.3 (Evolved Analytics LLC, Rancho Santa Fe, CA, USA) which runs on Mathematica version 12.1 (Wolfram Research Incorporated, Champaign, IL, USA). The explanatory variables included 34 pre-operative variables (Supplementary Table 4) such as gender, age, height, body weight, blood pressure, smoking history, disease history, laboratory data, and CT volumetry data. DataModeler was executed with 8 independent evolutions and 6 min modeling time. During the evolution, SR via GP automatically discards less important variables and selects more important ones, effectively performing dimensionality reduction. Then it generates hundreds of predictive models, selects a few to a few dozen of simpler and less erroneous models, and uses their trimmed mean as the optimized predictive model, just like a bagging method used in random forest. The optimized model created through the process is referred to as “optimized donor’s kidney function (DKF) model” in this paper.

We also explored the driver variables in the optimized DKF model and their impact on the overall model, illustrating their effects on the target using a partial dependence estimate, based on the approach suggested by Friedman^32^ A partial dependence estimate calculates the average prediction of a model by fixing a feature at a specific value while averaging over the other features. This gives an estimate of how the feature of interest affects the model’s output. By holding the other features constant, we can understand the marginal effect of just the one feature.

We developed a simplified prediction formula from the optimized DKF model to enhance user-friendliness in clinical settings, where variables with smaller effects on the target were fixed at a representative value (median or mode). This model is referred to as the “sparse DKF model.”

As a conventional method for estimating renal function post-donation, a formula 0.7 × [eGFR at pre-donation] was used and is referred to as the “conventional DKF model”. This assumption is based on the previous report that post-donation eGFRs are typically reach approximately 60–70% of the pre-donation levels^4^.

### Verification of accuracy of developed models

To compare the accuracy among the optimized DKF model, the sparse DKF model, and the conventional DKF model, metrics such as R^2^, root mean squared error (RMSE), mean absolute error (MAE), and Pearson’s correlation coefficient between each predicted value and measured eGFR were utilized.

Additionally, outliers in the optimized DKF model were identified using Z-scores, which represent the distance of a data point from the mean in terms of standard deviations. The standard cutoff values for defining outliers were Z-scores of ± 2 or more extreme. Based on the Z-scores, the validation cohort was divided into three groups: inliers (-2 < Z < 2), high outliers (Z > 2), and low outliers (Z < -2). This classification was used to investigate preoperative factors that are likely to contribute to outliers.

## Electronic supplementary material

Below is the link to the electronic supplementary material.

Supplementary Material 1

## Data Availability

The data which support the findings of this study are available from the corresponding author, [T.H.], upon reasonable request. The generated model by machine learning was published in GitHub (https://github.com/thirai-0813/Donor_Cr_Calculator_EN.git).
