# Supplementary material for: Personalized prediction model generated with machine learning for kidney function one year after living kidney donation
Source: Sci Rep. 2025 Jul 1;15:20752. doi: 10.1038/s41598-025-02879-y (PMC12215664; doi:10.1038/s41598-025-02879-y)
Supplement: Supplementary file 1 — Supplementary Material 1 [file 41598_2025_2879_MOESM1_ESM.pdf]

Supplementary Tables

Supplementary Table 1 (A). Correlation between CT volumetry data and preoperative creatinine level.

Variables	r (95% CI)	*p*
Volume of excised kidney	0.15 (0.02--0.29)	0.029
Volume of non-excised kidney	0.15 (0.02--0.28)	0.035
Area psoas muscle at L3	0.29 (0.16--0.41)	<0.001
Area skeletal muscle at L3	0.65 (0.57--0.73)	<0.001
Area visceral fat	0.36 (0.23--0.47)	<0.001
Area visceral Fat at L3	0.45 (0.33--0.55)	<0.001

Supplementary Table 1(B). Correlation between CT volumetry data and preoperative body weight.

Variables	r (95% CI)	*p*
Volume of excised kidney	0.60 (0.50--0.69)	<0.001
Volume of non-excised kidney	0.63 (0.54--0.71)	<0.001
Area psoas muscle at L3	0.34 (0.21--0.46)	<0.001
Area skeletal muscle at L3	0.86 (0.82--0.89)	<0.001
Area visceral fat	0.64 (0.55--0.71)	<0.001
Area visceral Fat at L3	0.69 (0.61--0.75)	<0.001

Supplementary Table 2. The comparison of patient characteristics between inlier (-2<Z<2), high outlier (2<Z), and low outlier group (Z<-2) in validation cohort for predicting creatinine at 1 year post-donation. (The cases with outliers matched between the optimized DKF model and the conventional DKF model)

Variables	-2<Z<2 (*N*=58)	2<Z (*N*=1)	Z<-2 (*N*=2)	*p*
Age at donation (y.o)	58.8±9.00	54.8	68.3±4.85	NA
Male [n (%)]	17 (29.3)	0 (0)	1 (50)	0.67
Body weight (kg)	53.0 (50.3, 61.5)	53.0	59.5 (58.8, 60.3)	0.49
Height (cm)	159 (154, 165)	160	158 (158, 159)	0.92
BMI	21.9 (20.2, 23.1)	20.7	23.7 (23.3, 24.1)	0.28
Systolic blood pressure (mmHg)	128.2± 20.6	105	130.5±3.54	NA
Diastolic blood pressure (mmHg)	75.1±17.3	62	81.0±8.49	NA
smoking [n (%)]	15 (25.9)	0 (0)	1 (50)	0.62
History of CVD [n (%)]	1 (1.7)	0 (0)	0 (0)	0.97
Antihypertensive agents [n (%)]	12 (20.7)	0 (0)	1 (50)	0.53
Lipid-lowering agents [n (%)]	8 (13.8)	0 (0)	1 (50)	0.33
uric acid lowering agents [n (%)]	0 (0)	0 (0)	0 (0)	NA
WBC (/μl)	5240 (4283, 6248)	3960	4165 (3653, 4678)	0.18
Hb (g/dl)	13.7±1.18	12.3	13.7±1.41	NA
Platelet ($*10^3$/μl)	21.5 (18.5, 26.5)	17.1	15.1 (13.8, 16.4)	0.09
Cr (mg/dl)	0.69 (0.62, 0.77)	0.52	1.07 (1.01, 1.12)	0.03
BUN (mg/dl)	13.8 (11.4, 15.6)	12.4	16.5 (16.0, 16.9)	0.30
Na (mEq/L)	141(140, 143)	144	142	0.29
K (mEq/l)	4.2 (4.0, 4.4)	3.8	4.3 (4.2, 4.3)	0.40
AST [IU/l]	20.0 (17.0, 23.8)	31	21.5 (20.8, 22.3)	0.28
ALT [IU/l]	16.5 (13.3, 22.8)	43	17.0 (16.5, 17.5)	0.26
TP (g/dl)	7.24± 0.34	7.20	7.30± 0.57	NA
CRP (mg/dl)	0.05 (0.04, 0.11)	0.05	0.05 (0.04, 0.06)	0.83
T.chol (mg/dl)	206 (190, 236)	193	188 (185, 190)	0.38
UA (mg/dl)	4.7 (4.1, 5.9)	3.0	5.7 (5.2, 6.1)	0.22
Glucose (g/dl)	98 (91, 106)	93	112 (104, 119)	0.52
HbA1C (%)	5.7 (5.5, 5.9)	5.9	5.6 (5.5, 5.6)	0.38
Proteinuria (+)	1 (1.7)	0 (0)	0 (0)	0.97
Urinary occult blood (≥ +)	3 (5.2)	0 (0)	0 (0)	0.92

**Results of CT findings**				
Volume of excised kidney (ml)	142(118, 157)	128	122 (117, 127)	0.41
Volume of non-excised kidney(ml)	135 (114, 152)	144	115 (114, 115)	0.45
Area psoas muscle at L3(cm^2)	10.7 (8.8, 15.1)	9.3	16.9 (15.0, 18.9)	0.27
Area skeletal muscle at L3(cm^2)	92.1 (82.1, 118)	88.7	132 (128, 136)	0.23
Area visceral fat(cm^2)	88.5 (49.0, 116)	90.2	110 (102, 118)	0.65
Area visceral Fat at L3 (cm^2)	83.1 (38.0, 118)	75.4	123 (90.8, 155)	0.74

Continuous data are presented as mean ± SD or median (IQR): CVD, cardiovascular disease; WBC, white blood cell; Hb, hemoglobin; Cre, creatinine; eGFR, estimated glomerular filtration rate; BUN, blood urea nitrogen; Na, sodium; K, potassium; AST, aspartate aminotransferase; ALT, alanine aminotransferase; TP, total protein; CRP, C-reactive protein; T.chol, total cholesterol; UA, uric acid; HbA1C, hemoglobinA1C

Supplementary Table 3.

A. Japanese criteria for living donor kidney transplantation

Age	≥ 20 years old and ≤ 70 years old
Obesity	≤ 30 kg/m2
Renal function	GFR≥ 80ml/min/1.73m2
Urinalysis abnormality	u-pro<150mg/day or 150mg/gCr, and albuminuria <30mg/gCr
Hypertension	BP <140/90 mmHg
Diabetes	FBS≤126 mg/dl and HbA1C<6.2%

B. The marginal criteria for living kidney transplantation in Tokyo women's medical university

Age	≥ 20 years old and ≤ 80 years old
Obesity	≤ 25-30 kg/m2
Renal function	GFR≥ 70ml/min/1.73m2
Urinalysis abnormality	u-pro<150mg/day or albuminuria <30mg/gCr
Hypertension	BP <140/90 mmHg or <130/80 mmHg under antihypertensive agent
Diabetes	FBS≤126 mg/dl and HbA1C<6.2% (oral hypoglycemic agent is not allowed.)

GFR, glomerular filtration rate; u-pro, urinary protein; BP, blood pressure; FBS, Fasting Blood Sugar; HbA1C, hemoglobinA1C

Supplementary Table 4.

Variables
Age at donation (y.o)
Male
Body weight (kg)
Height (cm)
Systolic blood pressure (mmHg)
Diastolic blood pressure (mmHg)
smoking
History of CVD
Antihypertensive agents
Lipid-lowering agents
uric acid lowering agents
WBC (/μl)
Hb (g/dl)
Platelet ($*10^3$/μl)
Cre (mg/dl)
BUN (mg/dl)
Na (mEq/L)
K (mEq/L)
AST [IU/l]
ALT [IU/l]
TP (g/dl)
CRP (mg/dl)
T.chol (mg/dl)
UA (mg/dl)
Glucose (g/dl)
HbA1C (%)
Proteinuria (+)
Urinary occult blood (≥+)
**Results of CT findings**
Volume of excised kidney (ml)
Volume of non-excised kidney(ml)
Area psoas muscle at L3(cm^2)
Area skeletal muscle at L3(cm^2)
Area visceral fat(cm^2)
Area visceral Fat at L3 (cm^2)

CVD, cardiovascular disease; WBC, white blood cell; Hb, hemoglobin; Cre, creatinine; eGFR, estimated glomerular filtration rate; BUN, blood urea nitrogen; Na, sodium; K, potassium; AST, aspartate aminotransferase; ALT, alanine aminotransferase; TP, total protein; CRP, C-reactive protein; T.chol, total cholesterol; UA, uric acid; HbA1C, hemoglobinA1C

Supplementary documentation

Supplementary documentation 1

The estimated formula for creatinine level at 1-year post-donation is shown below.

The selected factors for formula are age, male, BW, the history of CVD, BUN, Cre, HbA1C, and volume of non-excised kidney. The MedianAverage is equivalent to trimmed mean, which is the average of a dataset after removing a specified percentage of the highest and lowest values to reduce the impact of outliers.

$$
\begin{aligned}
\mathrm{MedianAverage}\Bigg[\Bigg\{ & -0.09903755662234529 + 0.006502924180113298\mathrm{Bw} \\
& + 1.3625035862329375\sqrt{\mathrm{Cre}} + 0.1139767991517405\mathrm{Male} \\
& - 0.0027455075816872963\mathrm{NonExcisedKidney}, -0.3901364879414445 \\
& + 0.04471756216946074\mathrm{BUN} + 0.006715659519617908\mathrm{Bw} \\
& + 1.0352518798689898\mathrm{Cre} \\
& - 0.000009604315179117748\mathrm{BUN}^2\boxtimes\mathrm{NonExcisedKidney}, -0.38478682583687274 \\
& + 0.002081547696286225\mathrm{Age} + 0.04399956263153849\mathrm{BUN} \\
& + 0.005628920758504391\mathrm{Bw} + 0.9320131800396743\mathrm{Cre} \\
& + 0.06722065750493375\mathrm{Male} \\
& - 0.000009867424180159245\mathrm{BUN}^2\boxtimes\mathrm{NonExcisedKidney}, 0.049617816124754036 \\
& + 0.9225151798784034\mathrm{Cre} + 0.18320900637720863\mathrm{CVD} \\
& + 0.10349178758374927\mathrm{HbA1C} + 0.13985579153513217\mathrm{Male} \\
& - 0.0019323076033121343\mathrm{NonExcisedKidney}, -0.18899026587394271 \\
& + 0.024398835894397737\mathrm{BUN} + 0.0032842131730298754\mathrm{Bw} \\
& + 1.142965718977502\mathrm{Cre} \\
& - 9.754126416415309 \times 10^{-9}\mathrm{BUN}^4\boxtimes\mathrm{NonExcisedKidney}, -0.006961262066861628 \\
& + 0.8126766126098913\mathrm{Cre} + 0.11057326356803435\mathrm{Male} \\
& + \frac{1.4573284728625062}{0.7326765340341233 + \frac{\mathrm{NonExcisedKidney}}{\mathrm{Bw}}}, 0.005082859737290714 \\
& + 0.03531000856642338\mathrm{BUN} + 0.00699894215253158\mathrm{Bw} \\
& + 0.7899600124990199\mathrm{Cre} + 0.10910119812549417\mathrm{Male} \\
& - 0.0002167791209982864\mathrm{BUN}\boxtimes\mathrm{NonExcisedKidney}, 1.2549512514779957 \\
& + 0.005164327440285673\mathrm{Bw} + 0.8188362941962077\mathrm{Cre} - \frac{4.127069601506772}{\mathrm{CVD} + \mathrm{HbA1C} + \mathrm{Male}} \\
& - 0.0026704663767301066\mathrm{NonExcisedKidney}, -0.15903717053578167 \\
& + 0.00025977004836070643\mathrm{Age}\boxtimes\mathrm{BUN} + 0.005291867117086743\mathrm{Bw} \\
& + 1.0913423662894317\mathrm{Cre} \\
& - 6.322735119054266 \times 10^{-12}\mathrm{BUN}^3\boxtimes\mathrm{NonExcisedKidney}^3\Bigg\}, \frac{1}{9}
\end{aligned}
$$

BW, body weight; CVD, cardiovascular disease; BUN, blood urea nitrogen; Cre, creatinine; HbA1C, hemoglobinA1C

Supplementary Figures

Supplementary Figure 1. Correlation chart ranked by correlation coefficient

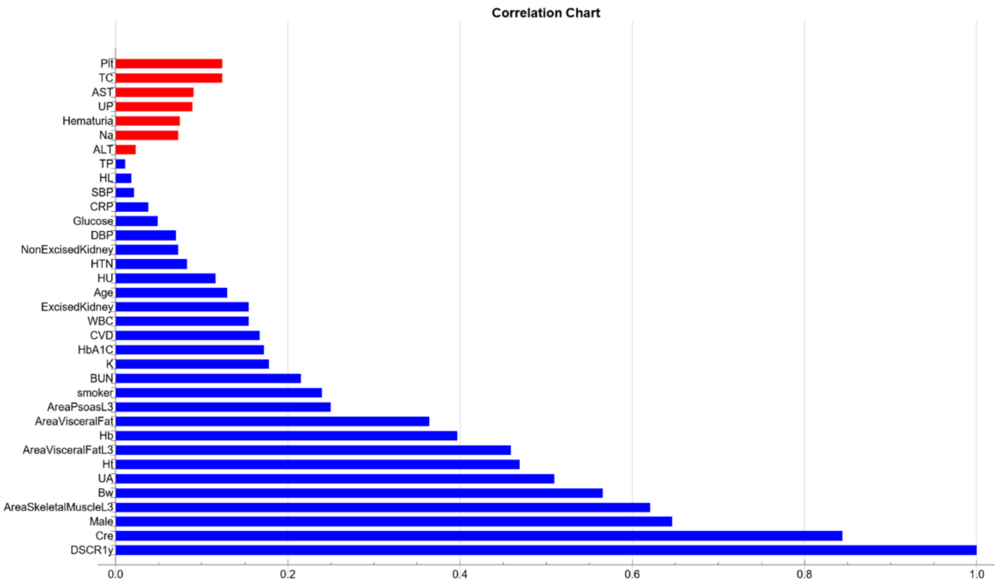

correlation coefficient

The blue bars represent positive correlations, while the red bars represent negative correlations.

Plt, platelet; TC, total cholesterol; AST, aspartate aminotransferase; UP, urine protein; Na sodium; ALT, alanine aminotransferase; TP, total protein; HL, hyperlipidemia (lipid-lowering agents); SBP, systolic blood pressure; CRP, C-reactive protein; DBP, Diastolic blood pressure; HTN, hypertension; HU, hyper uricemia (uric acid lowering agents); WBC, white blood cell; CVD, cardiovascular disease; HbA1C, hemoglobinA1C; K, potassium; BUN, blood urea nitrogen; Hb, hemoglobin; Ht, height; UA, uric acid; Bw, body weight; Cre, creatinine; DSCR1y; serum creatinine of donor at 1-year post-donation
The X-axis represents the correlation coefficient.

Supplementary Figure 2. Impact of the top three variables on partial dependence estimate in the optimized DKF model

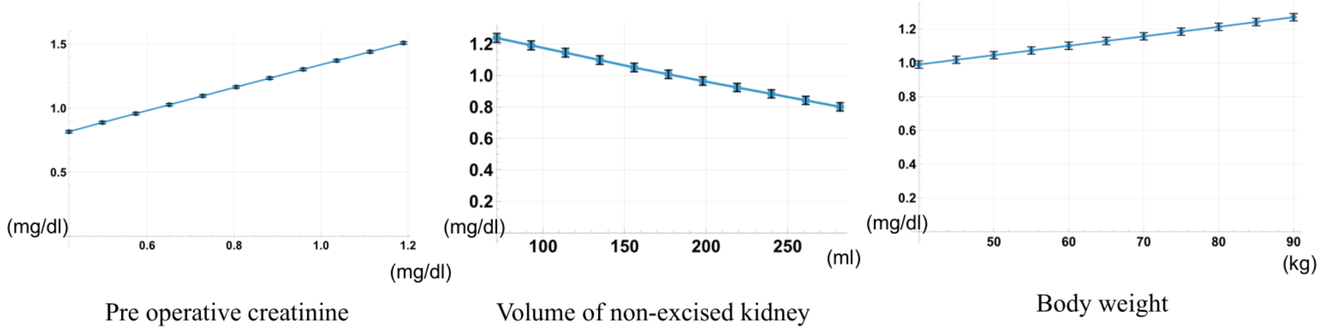

Partial dependence estimate represents the change in the target variable when each explanatory variable is varied from its minimum to its maximum, while the influence of all other variables was averaged out. Preoperative creatinine, the volume of the non-excised kidney, and body weight were identified as the top three contributors to partial dependence estimate for predicting creatinine levels at 1 year post-donation. The X-axis represents the range of the target variable from its minimum to maximum value. The Y-axis represents the predicted value of creatinine levels at 1 year post-donation.

Supplementary Figure 3. The representative CT images for volumetry

(A) (B) (C)

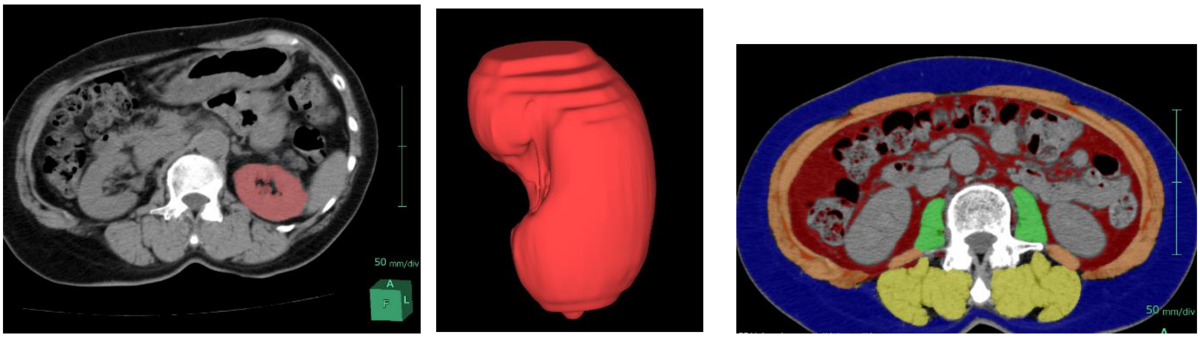

(A) An axial plane of the nephrographic phase. The kidney outline is semiautomatically created by grouping pixels with identical CT values on the axial plane.

(B) A 3D reconstructed image generated automatically.

(C) Each color represents the following areas: Blue indicates subcutaneous fat, Red indicates visceral fat at the navel level, Green indicates the psoas muscle, Orange indicates abdominal muscles, and Yellow indicates erector spinae muscles. The skeletal muscle area is represented as the sum of the psoas major, abdominal muscles, and erector spinae muscles.
